# Serum Creatinine Level in Relation to Intraluminal Thrombus and Abdominal Aortic Aneurysm Size

**DOI:** 10.3390/jcm14041258

**Published:** 2025-02-14

**Authors:** Louise Røtzler Holm, Jonas Peter Eiberg, Qasam M. Ghulam, Alexander Hakon Zielinski, Rebecca Andrea Conradsen Skov, on behalf of the COACH Research Collaborative

**Affiliations:** 1 Department of Vascular Surgery, Rigshospitalet, 2100 Copenhagen, Denmarkjonas.peter.eiberg@regionh.dk (J.P.E.); alexander.hakon.zielinski@regionh.dk (A.H.Z.); 2 Department of Clinical Medicine, Faculty of Health and Medical Sciences, University of Copenhagen, 1172 Copenhagen, Denmark; 3 Copenhagen Academy for Medical Education and Simulation (CAMES), 2100 Copenhagen, Denmark

**Keywords:** abdominal aortic aneurysm, intraluminal thrombus, creatinine, ultrasound, biomarker

## Abstract

**Objectives**: Abdominal aortic aneurysm (AAA) diameter is the primary predictor of AAA rupture. However, smaller aneurysms do rupture, and other parameters are required for a more nuanced risk stratification. Reduced renal function is associated with increased cardiovascular risk and thrombosis, but the impact of renal function on ILT and AAA size remains unknown. This study aimed to investigate the association between creatinine level and volume of ILT and AAA. **Methods**: In a cross-sectional study, 184 patients with AAA under ultrasound surveillance were included. ILT volume and thickness, and AAA volume and diameter, were measured using three-dimensional contrast-enhanced ultrasound. ILT and AAA measures were compared with creatinine levels. **Results**: No associations were found between creatinine level and ILT or AAA volume (*p* = 0.18 and *p* = 0.41). There were no differences in ILT volume between patients with normal and elevated creatinine levels, when adjusting for AAA size and comorbidities (*p* = 0.06 and *p* = 0.54). A positive association was found between ILT volume and AAA volume (*p* < 0.001). Creatinine level did not influence this association (*p* = 0.06). **Conclusions**: In this study, creatinine level did not seem associated with ILT or AAA volume. Longitudinal studies are required to elucidate associations between renal function, clinical outcomes, and ILT and AAA development.

## 1. Introduction

An abdominal aortic aneurysm is a dilation of the abdominal aorta of at least 3 cm [1,2,3]. Most patients with an abdominal aortic aneurysm are asymptomatic, but in case of rupture, the overall mortality is approximately 80% [1,2,3]. Consequently, patients with abdominal aortic aneurysms are offered elective repair to prevent rupture when the aneurysm diameter reaches 5.5 cm in men and 5 cm in women, if the maximum diameter increases more than 1 cm in a year, or if the aneurysm becomes symptomatic, for example, abdominal pain [1,2,3].

Patients with a small abdominal aortic aneurysm are recommended to enroll in an ultrasound surveillance program to monitor the potential growth of the aneurysm [1,2]. Ultrasound is ideal for surveillance due to its low costs, no use of radiation or nephrotoxic contrast compared to Computed Tomography (CT), and due to the possibility of handling the ultrasound scan in the outpatient clinic [1,2,3,4]. Maximum abdominal aortic aneurysm diameter is the most used criterion to determine the risk of rupture [1,2]; however, smaller aneurysms do rupture [5,6]. Therefore, measures to improve risk stratification of patients with an abdominal aortic aneurysm are being investigated to reduce the number of ruptures [5,7].

One risk factor of interest is intraluminal thrombus. Studies investigating associations between intraluminal thrombus and aneurysm growth and rupture are, however, contradicting [5,7]. Some studies suggest that intraluminal thrombus can halt aneurysm growth rate and increase aortic wall stability due to reduced wall stress [8,9]. Other studies indicate that increased intraluminal thrombus burden leads to aortic wall hypoxia and is associated with increased growth rate and increased risk of rupture at smaller diameters [10,11,12]. Even though research on the role of intraluminal thrombus in abdominal aortic aneurysm growth and rupture is still ongoing, evidence suggests that intraluminal thrombus in some way is involved in aneurysm progression [7,10,11,12,13].

However, incorporating the presence and size of intraluminal thrombus in conventional two-dimensional ultrasound surveillance programs to obtain a more nuanced risk stratification is not possible. Conventional ultrasound underestimates the diameter of the abdominal aortic aneurysm and cannot detect the presence of intraluminal thrombus in most aneurysms, which is why alternative technologies are being investigated [14,15,16]. Three-dimensional ultrasound (3D-US) and three-dimensional contrast-enhanced ultrasound (3D-CEUS) can be used to measure intraluminal thickness and volume, as well as abdominal aortic aneurysm diameter and volume, noninvasively and without the use of radiation or nephrotoxic contrast [4,16]. Abdominal aortic aneurysm volume measurements, with 3D-US, are a novel approach that can supplement aneurysm diameter in the prediction of aneurysm rupture, as it is a more sensitive measure of size and may be a more accurate predictor of rupture [6,17], but is not yet available to all clinicians. Therefore, other indicators of intraluminal thrombus size would be beneficial when exploring new potential risk factors to include in future risk stratification strategies.

Many possible biomarkers have been investigated as prognostic factors for rupture, e.g., D-dimer, C-reactive protein (CRP), and matrix metalloproteinases, but none of these have proven predictive [2]. Chronic kidney disease is associated with peripheral arterial disease, and arterial and venous thrombosis [18,19]. Reduced renal function is associated with increased levels of inflammatory factors and endothelial dysfunction, which are believed to cause this higher prevalence of cardiovascular disease (CVD) [18]. An overexpression of coagulation markers, such as tissue factor, has been observed in patients with chronic kidney disease and is thought to contribute to the increased risk of thrombosis [20]. It could therefore be hypothesized that renal function could be used as a surrogate measure of intraluminal thrombus.

The aim of this study was to investigate a possible association between serum creatinine level, the size of the intraluminal thrombus, and the size of the abdominal aortic aneurysm.

## 2. Materials and Methods

### 2.1. Study Design

The presented study was an observational single-center cross-sectional study.

### 2.2. Patients

Patients from the Copenhagen Aortic CoHort (COACH) included from 1 January 2015 to 30 September 2020 were considered for the study. COACH is a single-center cohort of patients with asymptomatic infrarenal abdominal aortic aneurysms enrolled in an ultrasound surveillance program [16,21]. On time of inclusion in the cohort, demographic data were collected consisting of the following parameters: sex, age, body mass index (BMI), use of antithrombotic therapy and statins, smoking habits, and comorbidities including hypertension (defined as antihypertensive treatment), chronic obstructive pulmonary disease (COPD), stroke/acute myocardial infarction (AMI), and any type of diabetes.

### 2.3. Ultrasound Examination

Patients with an abdominal aortic aneurysm with a maximum diameter above 3 cm were eligible for COACH, and were examined with dual-plane ultrasound, 3D-US and 3D-CEUS with a 3D-Matrix transducer (X6-1 xMATRIX) on a Phillips EPIQ-7 US system. The maximum cross-sectional leading edge to leading edge abdominal aortic aneurysm diameter with dual-plane ultrasound was used to define eligibility for the cohort. The 3D-CEUS protocol has previously been described in detail [16]. Exclusion criteria were iliac or thoracic aneurysm and previous surgical treatment for abdominal aortic aneurysm.

For the 3D-CEUS examination, a bolus of 1.5 mL of ultrasound contrast (Sonovue, Bracco, Milan, Italy) was administrated through a catheter in the antecubital vein. Subsequently, the 3D-CEUS scan was performed with default contrast settings.

The 3D-US scans were processed offline in a semiautomatic segmentation software (AAA-prototype v.2.0, Philips Research, Suresnes, France) in two steps. First, the proximal and distal extensions of the aneurysm were manually defined with ring markers to mark the extent of the aneurysm. Secondly, the aneurysm wall was automatically delineated to establish the maximum aneurysm volume and diameter. It was possible to manually correct this outline in case the operator concluded the automatically generated outline was imprecise. The same steps were performed for the contrast-filled lumen. To obtain an estimate of the intraluminal thrombus volume, the volume of the contrast-filled lumen was subtracted from the total aortic aneurysm volume. The same calculations were performed for maximum intraluminal thrombus thickness [14,16,21]. The length of the centerline was fixed to 60 mm, to ensure the entire aneurysm was evaluated and to standardize the segmentations between operators. Patients were excluded if centerline length was below 60 mm, or if they had poor US image quality. This was carried out by categorizing each ultrasound scan into 1 of 3 classes defined as follows: Class 1, good quality was defined as a scan with visualization of the aorta both proximally and distally to the aneurysm in the longitudinal plane and visible contrast-bearing lumen in the cross-sectional and longitudinal plane with a well-defined aortic wall. Class 2, medium quality was defined as a well-defined aortic wall and contrast-bearing lumen in both longitudinal and cross-sectional planes, but the aorta proximally and distally to the aneurysm was poorly visualized. Class 3, poor quality, was defined as an aneurysm only visible in one plane and with poor visualization of the aorta proximally and/or distally to the aneurysm [16]. These manual segmentations were performed by three different operators. To ensure low inter-operator variability, 20 scans were performed together, to ensure consensus on how to delineate the aneurysm and contrast-bearing lumen. In a previous study on assessment of intraluminal thrombus volume with 3D contrast-enhanced ultrasound, intra- and inter-operator variability were shown to be low with a range of variability of +/−7.5 mL and +/−8.8 mL, respectively [16].

### 2.4. Serum Creatinine Level

Before injection of the contrast bolus, blood samples for creatinine assessment were drawn from the same catheter for later analysis. Serum creatinine level varies with age, sex, BMI and race, but, in general, a serum creatinine level below 110 micromol/L in men and 90 micromol/L in women is considered normal [22,23]. Based on this, creatinine levels were divided into two groups: normal (below 110 micromol/L) and elevated (above 110 micromol/L) for men, and normal (below 90 micromol/L) and elevated (above 90 micromol/L) for women. If their creatinine level was not assessed, patients were excluded from analyses.

### 2.5. Data Analysis and Statistics

Differences in intraluminal thrombus volume in patients with different levels of creatinine have not been investigated before; however, in a previous study investigating differences in intraluminal thrombus volume between patients on different antithrombotic therapies, a difference of 14 mL in volume was found [21].

Numeric data are presented as mean ± standard deviation if normally distributed or as median with interquartile range (IQR) if not normally distributed. Normality was tested with histograms and with QQ plots. Categorical data are presented as count (n) with percentages in brackets. Data were compared with one-way analysis of variance (ANOVA) if normally distributed and numerical and Fisher’s exact test for categorical data.

The relationship between creatinine level and both intraluminal thrombus and aortic aneurysm volume were investigated with a linear regression model, and the coefficients will be presented and visualized with scatterplots.

The median intraluminal thrombus and aortic aneurysm volume, and mean aortic aneurysm diameter and intraluminal thrombus thickness were compared between normal and elevated creatinine levels with ANOVA when normally distributed or Kruskal–Wallis test when not normally distributed. Analysis of covariance (ANCOVA) and a non-parametric ANCOVA were used to compare intraluminal thrombus volume and thickness between the groups while adjusting for aneurysm size, sex, age, comorbidities, smoking, and antithrombotic therapy.

All statistical analyses were performed in RStudio version 2022.12.0.353 (Posit Software, PBC, Boston MA, USA), and the statistical plan was completed with the guidance of a statistician.

### 2.6. Ethics

Prior to enrollment, patients were given written and verbal information about COACH, and they signed a consent form. The study protocol was approved by the local Ethical Committee of Copenhagen (H-6-2014-056).

## 3. Results

### 3.1. Patient Inclusion

**Figure 1:**
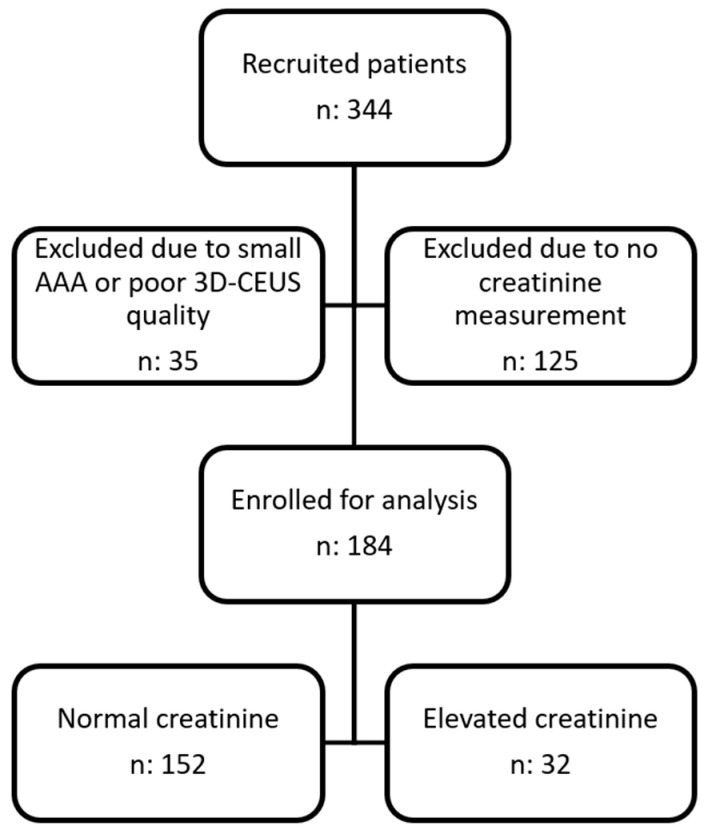
Flowchart of the 184 patients included in the study.

A total of 344 patients were included in COACH during the study period. A total of 35 patients were excluded due to a centerline length below 60 mm or poor imaging quality classified as class 3 as defined in the methods. Of the remaining 309 patients, 125 patients did not have their serum creatinine level determined leading to 184 patients being included in the study. The flowchart of inclusions and exclusions can be seen in Figure 1. The included patients were divided into two groups, according to their creatinine level: 152 (83%) patients had a normal creatinine level below 110 micromol/L, and 32 (17%) patients had an elevated creatinine level above 110 micromol/L.

### 3.2. Patient Demography

The mean age of the cohort was 73 (SD: ±7) years, and 82% of the cohort was male. A total of 31 (97%) patients with elevated creatinine levels were diagnosed with hypertension as compared to 106 (70%) of patients with normal creatinine levels (*p* = 0.003). In addition, six (19%) patients with elevated creatinine levels were diagnosed with diabetes as compared to eight (5%) patients with normal creatinine levels (*p* = 0.025). The remaining comorbidities were comparable, although more patients with elevated creatinine levels tended to receive anticoagulant therapy and statins (Table 1). Thirteen (9%) of patients with normal creatinine levels received anticoagulant therapy as compared to five (16%) (*p* = 0.49) of patients with elevated creatinine levels. A total of 113 (75%) of patients with normal creatinine levels received statins as compared to 31 (97%) (*p* = 0.011) of patients with elevated creatinine levels.

### 3.3. Creatinine Comparisons

**Figure 2:**
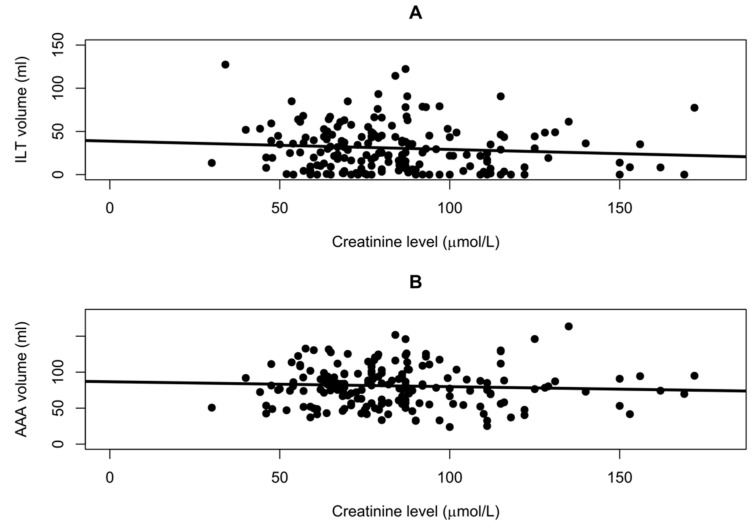
Linear regression analysis of (**A**): relation between serum creatinine level and ILT volume, and (**B**): relation between serum creatinine level and AAA volume. No effect of serum creatinine level on ILT volume of −0.10 mL/(μmol/L) was found (*p* = 0.18), and no effect of serum creatinine level on AAA volume of −0.07 mL/(μmol/L) was found (*p* = 0.41).

No association was found between creatinine level and intraluminal thrombus volume. Based on the linear regression analysis, when creatinine level increased by 1 micromol/L, the intraluminal thrombus volume decreased by 0.10 mL (*p* = 0.18) when not adjusting for comorbidities, antithrombotics and statins. When adjusting for these factors, an association was still not found. In addition, no associations were found between creatinine level and aortic aneurysm volume. In the unadjusted linear regression, the aneurysm volume decreased by −0.07 mL when creatinine increased by 1 micromol/L (*p* = 0.41). In the adjusted linear regression analysis, the aneurysm volume decreased by 0.12 mL when creatinine increased by 1 micromol/L (*p* = 0.19). To obtain a visual overview of these findings, a linear regression model demonstrating these can be seen in Figure 2. Figure 2A demonstrates the relationship between intraluminal thrombus volume and creatinine level. Figure 2B demonstrates the relationship between abdominal aortic aneurysm volume and creatinine level.

### 3.4. Intraluminal Thrombus and Aneurysm Size

The median intraluminal thrombus volume was 26 (IQR: 10–45) mL and 18 (IQR: 3–44) mL in patients with normal and elevated creatinine levels, respectively (*p* = 0.06). In the two groups, the mean intraluminal thrombus thickness was 12 ± 8 mm and 13 ± 7 mm, respectively (*p* = 0.65). The intraluminal thrombus volume and thickness compared with ANCOVA, and a non-parametric ANCOVA showed no differences between the groups (*p* = 0.06 and *p* = 0.54, respectively). The median aneurysm volume was 80 (IQR: 57–98) mL and 76 (IQR: 58–91) mL (*p* = 0.65), and the mean aneurysm diameter was 44 ± 7 mm and 42 ± 8 mm in patients with normal and elevated creatinine levels, respectively (*p* = 0.28). These results are outlined in Table 2.

### 3.5. Association Between Intraluminal Thrombus and Aortic Aneurysm

**Figure 3:**
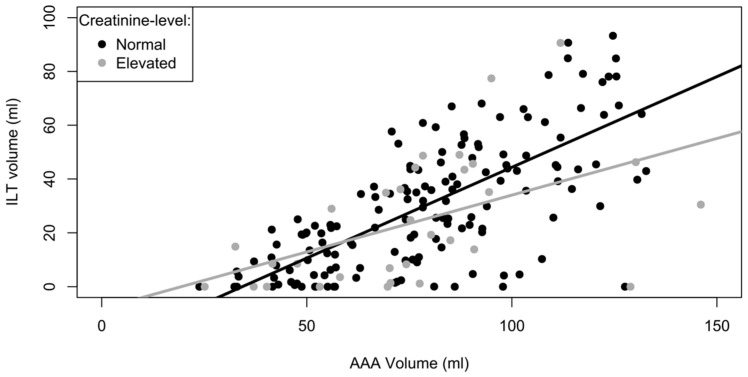
Linear regression analysis of the relation between ILT volume, AAA volume and serum creatinine level. A positive effect of AAA volume on ILT volume of +0.62 mL/mL was found (*p* < 0.001). The effect of creatinine level was insignificant (*p* = 0.07).

Figure 3 shows that the aneurysm volume and intraluminal thrombus volume are positively correlated with a coefficient of +0.62 mL/mL (*p* < 0.001). Creatinine level, however, did not affect this correlation between aortic aneurysm volume and intraluminal thrombus volume (*p* = 0.07). The same finding of no association to creatinine level was the case for the correlation between intraluminal thrombus thickness and aneurysm diameter.

## 4. Discussion

In this cross-sectional study, serum creatinine level was investigated as a potential biomarker for intraluminal thrombus volume and thickness, as well as for aortic aneurysm volume and diameter. No associations between creatinine level and intraluminal thrombus volume, intraluminal thrombus thickness, aortic aneurysm volume or diameter were found, but counterintuitively, a tendency towards lower thrombus load in patients with elevated creatinine levels was found. There was a positive correlation between intraluminal thrombus volume and aortic aneurysm volume.

Several studies have found a higher prevalence of chronic kidney disease in aortic aneurysm patients compared to control groups [24,25]. However, aortic aneurysm and chronic kidney disease share underlying risk factors such as older age, male sex, hypertension, and smoking habits, which may explain the co-existence of aortic aneurysm and chronic kidney disease [24,25]. A population screening study from 2018 demonstrated an association between low estimated glomerular filtration rate (eGFR) and the presence of aortic aneurysm and aortic aneurysm diameter [26]. Those findings contrast the results of this study but may be explained by using eGFR as a measure of renal function and conventional US for estimation of aortic aneurysm diameter.

Lindholt et al. examined 142 aortic aneurysm patients with US and measured serum creatinine [27]. They found no correlation between renal function (creatinine level and creatinine clearance) and aortic aneurysm diameter. Those findings are in line with another study that compared creatinine levels in 119 patients with small and large aortic aneurysm and found no differences [28]. However, none of these studies investigated aortic aneurysm volume which has been shown as a more subtle measure for aortic aneurysm changes [6,29]. Nevertheless, the results of the present study, using volumetric endpoints, support that creatinine is not a promising biomarker for aortic aneurysm size, be it diameter or volume.

Patients with CKD are at higher risk of both arterial and venous thrombosis [18,19]. Many pathophysiological mechanisms contribute to this increased thrombus formation as the kidneys, directly and indirectly, influence coagulation [30]. Several studies have presented an overexpression of coagulation markers in patients with CKD. In the case of kidney injury, an increase in uremic toxins has been demonstrated, and it is believed that these act as activators of tissue factor. Tissue factor activates the coagulation cascade and increases the risk of thrombosis [20,30]. Patients with CKD also have increased levels of pro-inflammatory factors, leading to increased levels of fibrinogen [30].

The kidneys regulate aldosterone release through the renin–angiotensin–aldosterone system. Increased aldosterone is associated with enhanced coagulation, reduced fibrinolysis, increased oxidative stress, and reduced nitric oxide levels leading to increased thrombus formation [30,31]. In addition, it has been demonstrated that CKD leads to inhibition of nitric oxide synthesis, increased platelet aggregation, and increased levels of von Willebrand factor [30,32]. Despite the evidence of a pro-thrombotic status in CKD patients, this study did not find an association between creatinine level and intraluminal thrombus volume, but surprisingly found a tendency of lower intraluminal thrombus volume in patients with elevated creatinine level. This finding could be explained by differences in use of antithrombotic therapies.

The role of intraluminal thrombus in aortic aneurysm progression is unclear. Intraluminal thrombus is formed in aneurysms when blood flow disturbances cause wall shear stress. Wall stress activates platelets and induces endothelial dysfunction leading to hemostasis and thrombus formation [8,9]. Some studies claim intraluminal thrombus reduces the mechanical stress of blood flow on the vessel wall, acting as a protective layer and thereby reducing rupture risk [8,9]. In contrast, several studies conclude that intraluminal thrombus increases rupture risk caused by weakening the aortic wall due to reduced oxygen flow and wall hypoxia [10,12]. In addition, intraluminal thrombus appears capable of producing enzymes that degrade the aortic wall, promoting rupture [8,33,34]. These contradicting results regarding intraluminal thrombus suggest intraluminal thrombus can be both protective or a risk factor depending on the size, composition, and morphology [7,8,9,13]. Studies suggest that intraluminal thrombus should be considered in risk stratification of aortic aneurysm patients, although more evidence is needed [11,33].

In this study, larger intraluminal thrombus volume was observed in larger aortic aneurysms. Other studies have observed a similar positive correlation between the size of intraluminal thrombus and aortic aneurysm size [10,21,34,35]. A prospective study examined patients with CT and found a significant increase in the aortic aneurysm diameter and intraluminal thrombus thickness over time along with a decrease in aortic wall thickness [35].

### Limitations

The present study entails several limitations worth mentioning. The present cross-sectional study cannot conclude causality between creatinine level and intraluminal thrombus size, or the opposite. For the same reason, the study does not determine clinical outcomes such as growth rate, rupture rate, elective repair, or mortality in relation to creatinine level. Even though no associations were observed between creatinine levels and the size of the intraluminal thrombus or aortic aneurysm, the data cannot exclude the possibility that creatinine levels might influence rupture risk. Future longitudinal studies are needed to address these gaps, providing deeper insights into the clinical relevance of renal function markers in aneurysm risk stratification and management.

In this study, serum creatinine level was used to measure renal function. GFR is the most accurate method for assessing kidney function [18]; however, creatinine level is used to calculate eGFR and is widely used as an estimate for renal function [22,23]. In addition, data on differences in muscle mass or dietary habits were not available and therefore not corrected for in the analyses, both factors that could impact creatinine levels.

The patients included in the study were predominantly males, which might limit generalizability of the study. In addition, only 32 patients had elevated creatinine levels which might have influenced the power of the study. The patients had various comorbidities that could influence intraluminal thrombus and aortic aneurysm size. On the other hand, this was adjusted for in the analyses and did not change the conclusions. The use of different antithrombotic therapies was considered a covariant as it impacts the size of the intraluminal thrombus [21]. The results from the ANCOVA were still insignificant when adjusting for these differences. However, compliance to the prescribed medications were not investigated.

Three-dimensional contrast-enhanced ultrasound has been validated for assessing aortic aneurysm and intraluminal thrombus size with results comparable to CT angiography [14,15,16]. Therefore, the risk of measurement bias in this study is believed to be low.

## 5. Conclusions

Serum creatinine level as a biomarker for intraluminal thrombus volume and abdominal aortic aneurysm volume is unlikely to be useful in future AAA risk stratifications. However, it remains unclear whether renal impairment might serve as a risk factor for the progression of abdominal aortic aneurysms and, consequently, the risk of rupture. While this study provides valuable insights, it does not definitively address the role of renal function in aneurysm outcomes. Based on the findings, further prospective studies are recommended to explore the relationship between renal function, aneurysm progression, and clinical outcomes, which could enhance understanding of disease mechanisms and improve patient risk stratification.

## Figures and Tables

**Table 1:** Characteristics of 184 patients with abdominal aortic aneurysm included in the study. Patients were divided in two groups according to their serum creatinine level.

	Total (n = 184)	Normal Creatinine (n = 152)	Elevated Creatinine (n = 32)	*p* Value
Male sex	151 (82)	123 (81)	28 (88)	0.53
Age, years	73 ± 7	73 ± 7	75 ± 6	0.11
BMI	27 ± 4	27 ± 4	27 ± 3	0.70
*Antithrombotic therapy*				0.46
None	22 (12)	18 (12)	4 (13)	
Antiplatelet	144 (78)	121 (80)	23 (72)	
Anticoagulant	18 (10)	13 (9)	5 (16)	
Statins	144 (79)	113 (75)	31 (97)	0.011
Antihypertensive medications	137 (75)	106 (70)	31 (97)	0.003
COPD	47 (26)	40 (26)	7 (22)	0.76
Stroke/AMI	63 (35)	52 (34)	11 (36)	1.00
Diabetes	14 (8)	8 (5)	6 (18.8)	0.025
*Smoking*				0.31
Currently	63 (34)	56 (37)	7 (23)	
Previously	105 (57)	84 (55)	21 (68)	
Creatinine level µmol/L	85.0 ± 27.0	75.0 ± 16.0	130.2 ± 22.0	<0.001

Data are presented as n (%) or mean ± standard deviation. Groups are compared with ANOVA or Fisher’s exact test. BMI = Body mass index; COPD = Chronic obstructive pulmonary disease; AMI = Acute myocardial infarction.

**Table 2:** Size of intraluminal thrombus and size of abdominal aortic aneurysm compared between patients with normal and elevated serum creatinine levels.

	Total (n = 184)	Normal Creatinine (n = 152)	Elevated Creatinine (n = 32)	*p* *	*p* ^¶^
ILT-volume, mL	25 (8–45)	26 (10–45)	18 (3–44)	0.06	0.06
ILT-thickness, mm	13 ± 7	12 ± 8	13 ± 7	0.65	0.54
AAA-volume, mL	78 (57–97)	80 (57–98)	76 (58–91)	0.65	-
AAA-diameter, mm	44 ± 7	44 ± 7	42 ± 8	0.28	-

Data are presented as n (%), mean ± standard deviation, or median (Interquartile range). AAA = Abdominal aortic aneurysm; ILT = Intraluminal thrombus; n = count. * ANOVA or Kruskal–Wallis test. ^¶^ Analysis of covariance (ANCOVA) parametric and non-parametric adjusting for aneurysm-size, sex, age, comorbidities, smoking and antithrombotic therapy.

## Data Availability

The data are not publicly available due to patient information.

## Appendix A

The COACH (the Copenhagen Aortic Cohort) Research Collaborative:

Ulver S. Lorenzen ^a,b^, Magdalena Broda ^a^, Marta Irene Bracco ^d,e^, Cecile Dufour ^d,^ Kim K. Bredahl ^a,c^, Laurence Rouet ^d^, Nikolaj Eldrup ^a,b^, Timothy Resch ^a,b^.

a Department of Vascular Surgery, Rigshospitalet, Copenhagen, Denmark

b Department of Clinical Medicine, Faculty of Health and Medical Sciences, University of Copenhagen, Denmark

c Copenhagen Academy for Medical Education and Simulation (CAMES), Denmark

d Philips Health Technology Innovation, Paris, France

e Mines Saint-Étienne, University Jean Monnet, INSERM, Sainbiose, Saint-Étienne, France
